# Analysis of Gender Issues in Computational Thinking Approach in Science and Mathematics Learning in Higher Education

**DOI:** 10.3390/ejihpe14110188

**Published:** 2024-11-08

**Authors:** Alejandro De la Hoz Serrano, Lina Viviana Melo Niño, Andrés Álvarez Murillo, Miguel Ángel Martín Tardío, Florentina Cañada Cañada, Javier Cubero Juánez

**Affiliations:** 1 Department of Experimental Science and Mathematics Teaching Area, University of Extremadura, 06006 Badajoz, Spain; lvmelo@unex.es (L.V.M.N.); andalvarez@unex.es (A.Á.M.); flori@unex.es (F.C.C.); jcubero@unex.es (J.C.J.); 2 Department of Computer and Telematic Systems Engineering, University of Extremadura, 06800 Merida, Spain; matardio@unex.es

**Keywords:** computational thinking, educational robotics, gender, science education, mathematics education, pre-service teachers

## Abstract

In the contemporary era, Computational Thinking has emerged as a crucial skill for individuals to possess in order to thrive in the 21st century. In this context, there is a need to develop a methodology for cultivating these skills within a science and mathematics content education framework, particularly among pre-service teachers. This study aimed to investigate the impact of Educational Robotics on the development of Computational Thinking skills, with a particular focus on the role of gender, through a scientific and mathematical content teaching approach. A pre-experimental design with a quantitative approach was employed, and it was implemented with a total of 116 pre-service teachers, 38 males and 78 females. The results demonstrated a notable enhancement between the pre-test (8.11) and post-test (9.63) scores, emphasising specific concepts such as simple functions, while, and compound conditional. With respect to gender, statistically significant differences were identified prior to the intervention, but not following its implementation. The high level of Computational Thinking exhibited by both genders was comparable (53.85% in females and 55.26% in males) following the intervention. This indicates that the intervention is a promising approach for enhancing Computational Thinking proficiency, independent of gender and initial proficiency levels. The implementation of Educational Robotics in the teaching of science and mathematics enables the enhancement of Computational Thinking abilities among pre-service teachers, while reducing the observed gender disparity in this area of skill development.

## 1. Introduction

Advances in computer science (CS), robotics, and artificial intelligence have become a primary factor in the development of early science and technology literacy learning [1,2]. In recent years, an increase in academic interest has been reported in the field of Computational Thinking (CT), which is perceived to align with the skills required in both the present and future societies. In response to this demand, the educational field must adapt to society based on technological, scientific, and mathematical development. It is, therefore, essential that individuals possess the requisite knowledge and skills to develop and participate in this field.

Concurrently, global attention is focused on the impact of gender in the scientific, mathematical, and technological domains, given the relatively low representation of women in these fields, which are experiencing a high degree of demand [3]. Prior research [4,5] indicates a need to consider the potential influence of gender on the learning process. This fact can be increasingly emphasised in a society based on science, technology, engineering, and mathematics (STEM), as findings indicate that attitudes and predispositions differ according to field or area of study [6].

In light of these considerations, there is a growing tendency to incorporate computer science (and thus, Computational Thinking) into disciplinary education, particularly in the domains of science and mathematics [7,8,9]. This approach is motivated by the recognition that these fields can offer valuable opportunities for CT learning [10,11]. However, the integration of CT into these disciplines remains a complex issue, as numerous practical challenges remain to be explored. These include the identification of effective activities and approaches, as well as the development of assessment strategies that are appropriate within the new context [9,12].

In accordance with this requirement, robotics education becomes progressively more integrated with STEM and CT practices [13]. To operationalise CT, researchers must consider a greater range of factors, including specific learning environments and learner characteristics. Prior research [14,15,16] indicates a necessity to expand training programs for pre-service teachers, with an emphasis beyond the primary and secondary education levels [17].

### 1.1. Computational Thinking

The definition of Computational Thinking (CT) has been subject to evolution since its initial appearance, particularly as a result of the contributions of Seymour Papert in the 1960s and most notably following the comprehensive formulation proposed by Wing [18]. In 2011, the International Society for Technology in Education (ISTE) and the Computer Science Teachers Association (CSTA) developed an initial comprehensive framework [19] with the objective of assisting educators in integrating Computational Thinking (CT) into their instructional practices. Computational thinking (CT) is defined as a problem-solving process that encompasses various components, including problem formulation, data organisation and analysis, data representation, abstraction, algorithmic development or generalisation, and the transfer of the problem-solving process.

Another pertinent framework, developed by Brennan and Resnick [20], is centred on the assessment of CT, based on their research in the domain of coding education. This framework identifies three essential dimensions and their respective elements. The dimensions include concepts (such as sequences, loops, parallelism, events, conditionals, operators, and data), practices (including incremental and iterative approaches, testing and debugging, reusing and re-mixing, and abstracting and modularising), and perspectives (expressing, connecting, and questioning).

Some international organisations, including the Computing at School Group, the Computer Science Teachers Association, and the Association for Computing Machinery, have developed guidelines to promote computational literacy among students. These guidelines emphasise the importance of teaching students the essential concepts and skills related to programming, such as algorithms, sequences, variables, conditionals, loops, synchronism, parallelism, procedures, and debugging. The objective is to enable students to develop solutions to concrete problems [1,3,13]. These findings are consistent with the conclusions of the 2016 European report on Computational Thinking, which defines Computational Thinking as a set of core competencies [3].

### 1.2. Computational Thinking Skills in Pre-Service Teachers

Teachers demonstrate a lack of knowledge, competence, self-efficacy, and self-confidence related to the effective integration of technology in the classroom and the utilisation of technology as a pedagogical tool [13,21]. The existing scientific literature has predominantly focused on the development of CT skills in K-12 students. Nevertheless, there is a paucity of evidence presented by teachers, particularly those in the pre-service classroom [22,23]. In order to enhance this area, the integration of development for pre-service teachers into curricula is recommended [16,23,24].

Yadav et al. [25] demonstrated that training methods and tools have a significant impact on the CT ability of pre-service teachers. Following a training program, there was a notable enhancement in the participants’ capacity for Computational Thinking (CT), as evidenced by their enhanced comprehension of CT principles, critical thinking abilities, innovation capabilities, and abstraction abilities. Indeed, research [26,27] has demonstrated that Educational Robotics (ER) is an effective instrument for cultivating Computational Thinking (CT) abilities at various stages of education, including higher education.

Despite the increase in the number of studies, further investigation is required to fill gaps in knowledge concerning pre-service teachers. Prior research [16,28] underscores the necessity for a comprehensive examination of the factors influencing the development of CT competencies, including strategies for integrating science and mathematics, as well as the impact of gender.

### 1.3. Influence of Gender on Computational Thinking Skills

Gender concerns have emerged as a significant area of study within the educational field, particularly given the growing evidence showing how stereotypes can have a profound impact on students’ attitudes and behaviours, and consequently on their learning experiences. Males are more predisposed to CS than females, while females require more time to complete tasks and develop CT skills [4,28]. Males tend to report higher levels of attitude and confidence, although females tend to demonstrate a superior performance in Computational Thinking skills. This situation was maintained across interventions in which female participants showed positive improvements in their self-efficacy, confidence, and programming skills [29]. The conventional gender role may play a significant role in shaping attitudes towards technology. However, this can be effectively modified under the appropriate conditions [4,9].

A study conducted by Esteve-Mon et al. [28] denoted that males revealed superior outcomes following the implementation of training in CT skills among a total of 114 pre-service teachers. Nevertheless, Angeli and Valanides [6] observed that girls can achieve greater progress in CT than boys through the implementation of collaborative programming practices. Meanwhile, Günbatar and Bakırcı [30] revealed how pre-service teachers did not vary based on gender, grade, or other limiting factors. It might be concluded that research on the comparative development of CT skills across genders still remains notably scarce. Tailored studies that examine these factors in the development of CT skills in pre-service teachers could lead to high a impact.

Additionally, it is highly remarkable that there is a reasonable lack of innovation and research on the integration of science and mathematics learning with Computational Thinking (CT) skills [9]. Based on the abovementioned situation, the authors consider that further investigation is required to fill this gap in the literature by analysing the influence of gender on the development of CT skills in pre-service teachers through science and mathematics content teaching approaches in pre-service teachers. In this manner, an appropriate methodology and the utilisation of adapted programming and ER resources can serve as invaluable tools for reducing gender disparities in the population [31,32].

### 1.4. Educational Robotics in Science, Mathematics and CT Skills

Educational Robotics (ER) is regarded as one of the most prominent topics by the international academic community. Several studies [14,17,33] have determined a high range of benefits provided by the development of students’ skills, with CT being a notable example. In light of the integration of technology, the learning by robotics approach might be considered as a highly effective methodology for Educational Robotics [15,16].

A rising number of studies are demonstrating the necessity for encouraging the development of CT skills across different disciplines, including science and mathematics [34,35]. The implementation of these skills might be initiated at the earliest educational stages, facilitating not only enhancements in CT capabilities, but also knowledge based skills in other subjects, such as science, mathematics, and engineering [36,37].

The inherent characteristics of these disciplines could be driven as a motivational practices in the professional world. In recent years, nearly every discipline associated with science and mathematics has shown a significant expansion based on the development of computer sciences, including bioinformatics, data analytics, computational statistics, chemometrics, and neuroinformatics [11]. Studies have indicated that the incorporation of CT into the domains of mathematics and science education [10] may offer certain benefits. In particular, CT enhances skills in topics such as the learning of complex scientific and mathematical concepts [38], particularly those that are aligned with the Next Generation Science Standards (NGSS) [9]. In 2018, the ISTE published the Standards for Educators. The Computational Thinking Competencies posit that CT skills can be developed and applied across all schoolages and subject areas. This assertion is also supported by prior research demonstrating how STEM activities boost CT skills, particularly those involving programming and ER [39].

Positive results in CT skills are achieved when ER is introduced with mathematics content [40,41,42]. This has also been demonstrated in studies conducted on students studying science content. Jaipal-Jamani and Angeli [43] and Sengupta et al. [10] presented significant increases in the self-efficacy and interest levels of their study samples. Alternatively, research conducted by Waterman et al. [44] and Gabriel-Le et al. [45] demonstrated that teachers enhanced their CT abilities and programming skills in the context of teaching science and mathematics based on a combined design, exhibiting a medium to high level of competence and developing a high sense of self-confidence in their teaching practices.

With respect to gender, there is evidence that females tend to hold more negative attitudes and predispositions than males regarding the use of programming and the learning of scientific and mathematical disciplines. Nevertheless, despite these negative perceptions, girls frequently demonstrate a greater academic proficiency compared to their male counterparts [4,5,6,46]. Nevertheless, ER has been demonstrated to possess significant potential for the mitigation of the gender disparities that have been observed in these disciplines [42,47].

Although research generally focuses on building teachers’ fundamental understanding of CT, only few studies have explored ways of enhancing their CT competency. Ye et al. [48] emphasised the necessity for studies that develop or support student learning and illustrated the lack of consensus among the research community on how to integrate the two fields. This suggests that the professional development of teachers in emerging competencies like scientific and mathematical thinking based on CT is critically important [1].

Considering these findings, the present study aims to analyse the Computational Thinking skills of pre-service teachers and the impact of the gender factor, examining changes before and after an intervention in the frameword of Educational Robotics and a science and mathematics approach. The following research questions will be addressed:

- RQ1: How does an ER-based intervention under a science and mathematics teaching approach influence pre-service teachers’ CT skills?
- RQ2: Does gender influence the CT skills of pre-service teachers before and after an ER-based intervention under a science and mathematics teaching approach?

## 2. Materials and Methods

### 2.1. Study and Participants

This study employed a pre-experimental design with a quantitative methodology that integrated both descriptive and inferential statistics. The research was performed as part of the university course entitled “Didactics of Mathematics I”.

It should be noted that the students on this course had not previously been exposed to programming elements during their undergraduate studies. The present research study took place in November and December of 2023. The participants received instruction in the area of Educational Robotics (ER) over the course of a five-hour training program. The study sample was selected for convenience, with a total of 116 participants. Of these, 38 were male and 78 were female. This study adhered to the principles of the Declaration of Helsinki [49].

### 2.2. Intervention

A block-based programming education program comprising five sessions (see Table 1), was implemented. In the initial session, the students were introduced to Scratch 3.0, a free software that employs block programming to facilitate the early acquisition of fundamental programming concepts. Specifically, the latter part of session one introduced it as a pedagogical tool for delineating and exploring geometric principles within the context of mathematical instruction at the primary education level.

The second session incorporated the “Mandala” challenge, where teams of four students created superimposed geometric figures to form a mandala. This task leveraged the software’s capacity to execute loops and create functions and conditionals, and prompted the participants to provide constructive feedback on the educational materials presented to them.

The third session focused on a theoretical explanation and experimentation with the possibilities offered by ER and the Mind Designer^®^ robotics kit. The participants had the opportunity to experiment with this resource by executing a series of programming tasks analogous to those previously described. The session concluded with a demonstration of activities designed to instruct in scientific and mathematical content. Subsequently, guidelines were provided for implementing Mind Designer^®^ in classroom activities for the development of instructional materials. After the presentation of the guidelines, the pre-service teachers were requested to develop a robotic board for use with primary school students, with the objective of facilitating instruction in the following scientific content: “*Teaching the healthy habit of hydration and its proper consumption*”.

**Figure 1:**
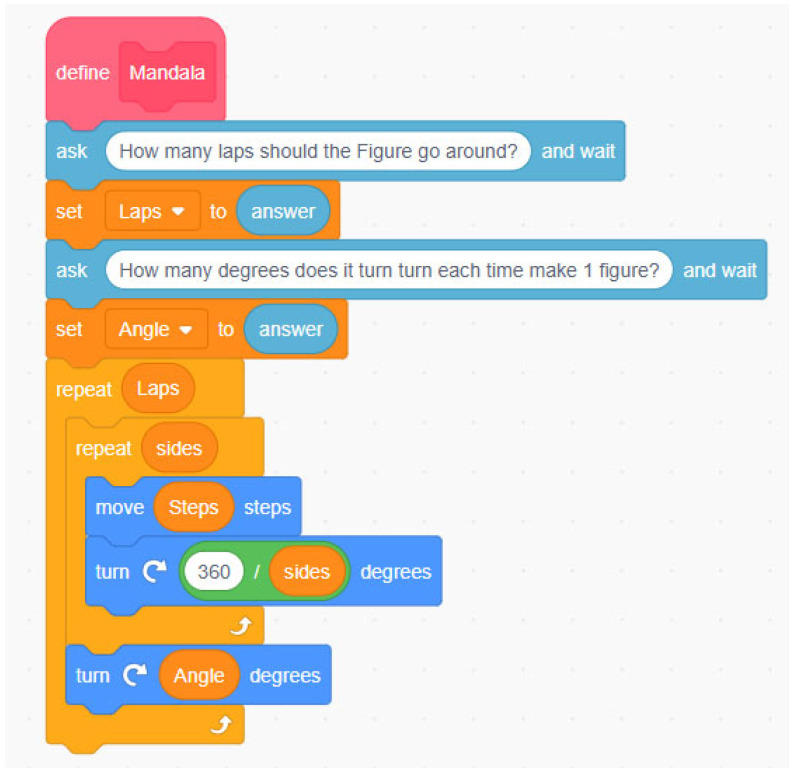
Example of programming sequence to create the mandala.

**Figure 2:**
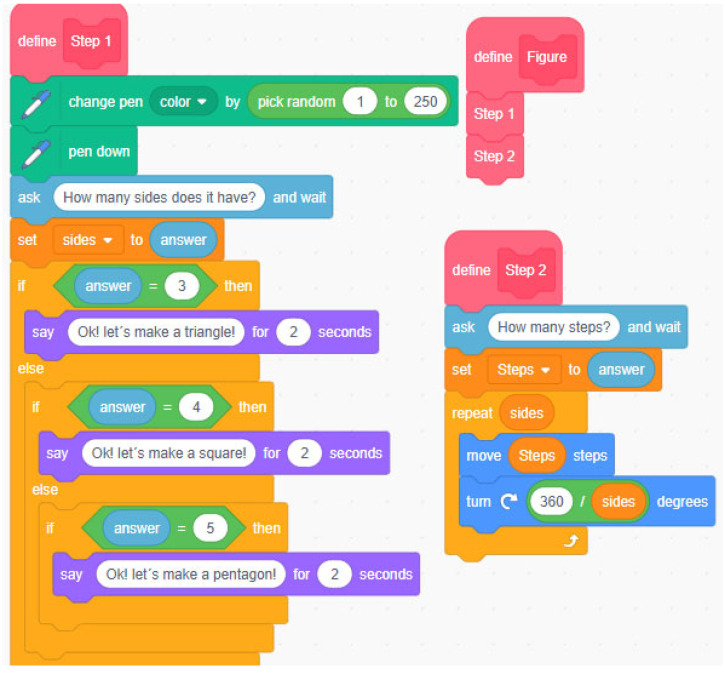
Example of a programming sequence for creating a geometric figure.

In the final two sessions, the students were assigned the task of elaborating on the proposed challenge, which included constructing a robotic board and preparing a report that detailed the teaching materials required. These included contextualisation, a description of the materials used, an account of the proposed activities or challenges, and guidelines for their implementation in a primary school classroom. Additionally, feedback was provided regarding the learning content. Figure 1 and Figure 2 illustrate examples of a programming sequence used by the students, showcasing various code blocks including conditionals, loops, functions (variables in Scratch), addresses, operators, and sensors

### 2.3. Measures Instruments

To measure the participants’ level of Computational Thinking, the Computational Thinking test (CTt) designed and validated by Román-González [50] was used. The CTt focuses on the following components: “sequences; loops; events; parallelism; conditionals; operators; data computational practices; problem-solving practices that occur in the process of programming; experimenting and iterating required task; testing and debugging; reusing and remixing; abstracting and modularizing” ([51], p. 679).

This questionnaire previously comprised 28 items; however, it was reduced to 14 items. These items address the various computational concepts that have been analysed, including *addresses, loops, conditional statements*, and *functions*. This reduction was implemented to align the questionnaire with the educational practice requirements. Four experts from the Department of Experimental Sciences and Mathematics Teaching at the university evaluated the instruments, assisted by two specialists in Telematics and Computer Engineering from the University of Extremadura. These individuals contributed to refining the wording and structure of each instrument.

Moreover, the CTt has been validated for use with a population of college students from a variety of academic backgrounds. In this regard, previous studies [52,53] have employed this questionnaire for the analysis of university students, with a particular focus on pre-service teachers in the field of primary education. Similar to these studies, the items were classified by complexity to align with the age of the students, and then validated using the appropriate statistical methods.

**Figure 3:**
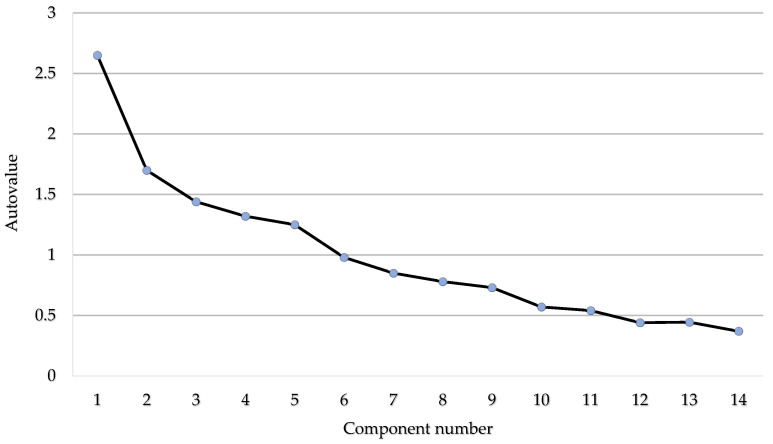
Sedimentation graph.

Additionally, a validation study was carried out with a sample with similar characteristics to the study group. To analyse the factorial structure of the selected items and verify the adequacy of the dimensions considered, a principal components factor analysis with oblimin rotation was performed. The Kaiser–Meyer–Oblin (KMO) index of sampling adequacy yielded a result of 0.603, while Bartlett’s test of sphericity confirmed a significant relationship [χ² (91 gl) = 256.1130; *p* < 0.001]. The seven-component model, with eigenvalues exceeding one (<0.4), explained 72.8% of the variance and fit the theoretical dimensions considered (see Figure 3). In addition, the consistency of the instrument was evaluated by the means of the Kuder–Richardson coefficient (KR20), obtaining a value of 0.715 for the 14 questions. In each of the dimensions considered, the coefficient was higher than 0.69, indicating an acceptable reliability of the instrument. Furthermore, the Cronbach’s alpha for internal consistency (α) was 0.79, which can be considered as a good reliability [54].

### 2.4. Data Analysis

To determine the level of Computational Thinking and to identify any changes in scores based on both pre-test scores and genders [55], we categorised the scores based on the total scores of the questionnaire into the following three levels: low (0–4 points), medium (5–9 points), and high (10–14 points). This approach allowed us to analyse the data in a more comprehensive manner, considering not only the overall score, but also the specific distribution of scores across different levels. The frequencies and percentages of participants belonging to each level are described for both the pre-test and the post-test. Moreover, a comparison was conducted between the pre-test and post-test scores to identify any statistical differences between the participants’ scores at each level. This analysis aimed to determine whether participants who initially scored at a specific level would remain at that level or shift to a different level after the intervention. Furthermore, it should be noted that, in this study, means (Xs) and standard deviations (SDs) were utilised, rather than medians, in conjunction with effect sizes (ESs) derived from Rosenthal’s r method, to analyse the statistical tests.

The quantitative analysis of the data was conducted using R software [56], which was employed for both descriptive and inferential statistical analysis. Owing to the non-normality of the data, as indicated by the Kolmogorov–Smirnov and Levene tests (*p* < 0.05), non-parametric statistical methods were employed. The Mann–Whitney U test was applied to independent samples, and the Wilcoxon test was used for related samples.

## 3. Results

**Figure 4:**
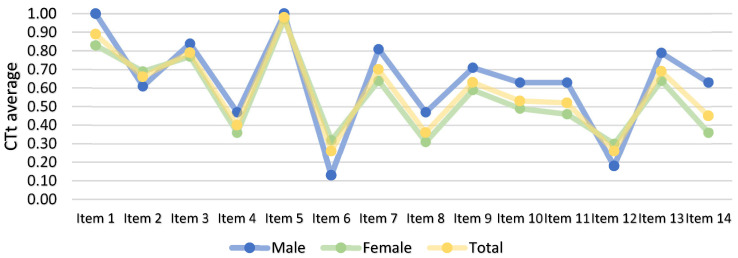
Descriptive results of each item of the pre-test questionnaire on Computational Thinking.

Table 2 and Figure 4 show the outcomes of the Computational Thinking Test (CTt) prior to the intervention. The results are presented on a global scale, as well as disaggregated by the participants’ genders. As evidenced by the data, the mean score for males was higher than that for females, and the standard deviation was lower for males.

Specifically, males demonstrated an elevated average for 11 of the 14 items on the questionnaire, while only items 2, 6, and 12 exhibited a higher mean for females. Regarding gender-related differences, it is notable that both male and female respondents exhibited a higher average number of correct answers for items 1 and 5 compared to the other items on the questionnaire. For these two items, the average number of correct answers was higher for males compared to females.

**Figure 5:**
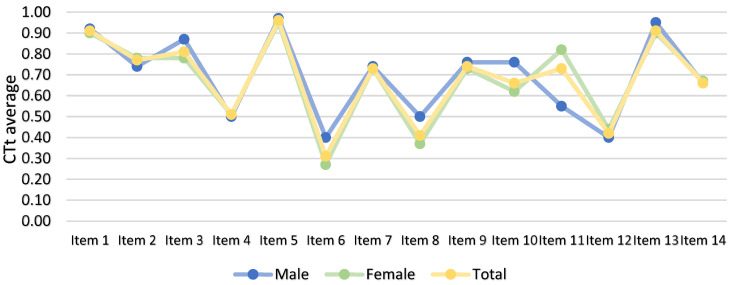
Descriptive results of each item of the post-test questionnaire on Computational Thinking.

A review of the results of the post-test, as detailed in Table 3 and Figure 5, reveals the descriptive results of the questionnaires administered after the intervention. These results are presented both globally and separately by gender. In this instance, the overall mean remained marginally higher for males, with minimal variation between genders.

A comparison of the items on the post-test reveals a greater disparity between genders. The male participant demonstrated a higher average on seven items, while the female participant demonstrated a higher average on five items. The remaining two participants exhibited similar averages. As can be observed in the post-test, the results for each item were notably closer between genders than they were in the pre-test, with the exception of item 11.

Table 4 illustrates the findings of the Mann–Whitney U test, which was conducted to identify any statistically significant differences between male and female participants in their pre-test and post-test results on the Computational Thinking Test (CTt).

The test revealed statistically significant differences (*p* < 0.05) in the pre-test, evident in the total score as well as in items 1, 6, and 14 specifically. Conversely, no statistically significant differences (*p* > 0.05) were evident in the total scores of the post-test results. However, a statistically significant difference was present for item 11.

Table 5 presents the findings of the Wilcoxon test, which examined the discrepancies between the pre-test and post-test results. The table displays the overall results, as well as the variations by participant gender.

The results demonstrate a statistically significant difference between the pre-test and post-test overall (*p* < 0.05). Additionally, items 4 and 9 exhibited indications of statistical significance, with values approaching *p* < 0.05. Upon examination of the data by gender, it was observed that there were statistically significant differences (*p* < 0.05) for female respondents, while there were no statistically significant differences for male respondents.

Upon closer examination of the data, it became evident that there were statistically significant differences (*p* < 0.05) for several items across different groups. Specifically, items 10, 11, 12, 13, and 14 showed statistically significant differences in the total results, while items 4, 11, 13, and 14 demonstrated such differences in the female group. Additionally, items 6 and 12 exhibited statistically significant differences in the male group. It should be noted that there were non-statistically significant values (*p* > 0.05) in both the *Repeat until* and *Simple conditional* sub-concepts in the total values.

**Figure 6:**
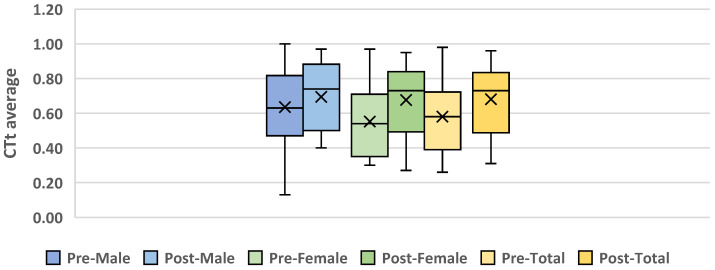
Box plot between pre-test and post-test of average total Computational Thinking scores.

Figure 6 presents a comparison of the pre-test and post-test outcomes, differentiated by gender and across the entire sample. As show in the box plots, the female respondents demonstrated a more significant increase in the mean score of the questionnaire following the intervention than the male respondents.

To ascertain the degree of Computational Thinking and to identify alterations in scores based on both the preliminary assessment scores and genders, as opposed to solely considering genders, Table 6 presents the frequencies and percentages of participants corresponding to each Computational Thinking level at both the preliminary assessment and post-assessment.

As illustrated, the low level showed a minimal percentage in both the pre-test and post-test. With regard to gender, it is evident that there was no representation of males in the pre-test. Conversely, the post-test included a single male participant. However, the proportion of female participants declined from 6.41% to 1.28%.

Notably, the medium level demonstrated a significant increase in representation prior to the intervention (71.55%) compared to the post-intervention period (43.97%). Specifically, the pre-test indicated a higher percentage of women than men, with 75.64% of women and 63.16% of men participating. In contrast, the post-test demonstrated very little difference in the percentages of women and men, with 44.87% of women and 42.11% of men involved.

Finally, with respect to the high level, an increase was observed between the initial assessment (24.14%) and the subsequent assessment (54.31%). Conversely, there was a notable divergence in the pre-test percentages between female (17.95%) and male (36.84%) subjects. In contrast, the post-test percentages exhibited a greater degree of similarity between the two genders (53.85% for females and 55.26% for males).

Table 7 presents the results of the Wilcoxon test, which examined the statistical differences between the pre-test and post-test averages for each level of participants. This analysis aimed to assess whether individuals initially grouped into a specific level in the pre-test remained in the same level or transitioned to a different one in the post-test.

At the high level, a decrease was observed in the results of the pre-test (11.63) and the post-test (10.00), with the overall mean remaining at a high level. In terms of gender, statistically significant differences were observed for the female participant (0.02), while the male participant showed indications of significance (0.07).

Finally, the low level displayed indications of significance (0.06), exhibiting an increase from 3.00 in the pre-test to 10.40 in the post-test, situated within the range of the high level. This result is consistent with the findings for females, given that no males participated in the pre-test and were, therefore, not included in the low-level category.

## 4. Discussion

The increasing demand for and importance of CS skills encourage educational institutions consider how to provide training programs to enhance professional knowledge and skills, including CT [31,57]. Research has shown that ER and the learning of science and mathematics can be beneficial for the development of CT [10,11]. However, there is a gap in the research regarding the influence of Educational Robotics interventions on the development of CT skills and the gender of pre-service teachers when they are focused on the teaching of science and mathematics content [25,41,42]. Our study attempts to provide scientific support for methodological strategies that allow for incorporating ER in the context of teaching scientific and mathematical content to develop CT skills in pre-service teachers.

In answering the first research question, *how does an ER-based intervention under a science and mathematics teaching approach influence the CT skills of pre-service teachers?*, the results of the intervention showed an increase in CT skills among the total scores between the pre-test (8.11 ± 2.52) and post-test (9.63 ± 2.75). Table 5 shows that these differences were statistically significant, which is consistent with previous studies [27,43,52]. Specifically, statistically significant differences were observed in subconcepts such as *Simple functions* and *While*, in which both items presented these statistically significant differences. In addition, there were also statistically significant differences in item 10, which belongs to the sub-concept *Compound conditional*. It was also possible to appreciate signs of statistical significance (*p*~0.05) in items 2, 4, and 9.

Analysing the descriptive results (Table 2 and Table 3), in most items where no statistically significant differences were found, there were already high pre-test means, which explains that the main differences in CT skill development were related to more complex concepts and subconcepts. Thus, the intervention and approach of this study allowed for preservice teachers working with ER related to the teaching of science and math content to develop certain complex CT skills. Nevertheless, we need to consider as a limitation in this research the existence of a lack of learning in some CT skills, such as *Repeat until* and *Simple Condition*, due to the fact that both items of each computational subconcept presented a statistical value far from being significant. Items 6 and 8 presented a not very high average in the pre-test that did not increase much after the intervention. For this reason, despite the great results obtained, it is necessary to improve the strategies for training these skills in future training programs, as well as to continue with further studies with the intention to understand the influence of this resource on training all the sub-concepts of CT.

In general, these results were consistent in regard to the different levels categorised for Computational Thinking (Table 6), in which it was observed that the percentage of the sample who belonged to the high level increased significantly, going from 24.14% to 54.31%. In contrast, the low and medium levels decreased significantly after the intervention, from 4.31% in the low level and 71.55% in the medium level in the pre-test to 1.72% and 43.97% in the post-test. This indicates that the participants improved their CT skills, with the ER intervention being effective despite the level at which they started.

Participants belonging to a particular CT level in the pre-test (Table 7) remained in the same level after the intervention. In the medium level there were statistically significant differences between the pre-test (7.24 ± 1.35) and the post-test (9.47 ± 1.35). This means that the sample with a medium level in the pre-test continued to belong to this level in the post-test, although the average was very close to the high level, so with a larger sample it could be expected to reach the high level. In the low level, although there were no statistically significant differences, it presented a value very close to being significant (*p* = 0.06), finding an increase from 3.00 (±0.71) to 10.40 (±2.07). In this case, it should be noted that the sample size was small (n = 5) at this level, so it would be expected that a larger sample size would have a greater impact on these results. Nevertheless, this increase can be appreciated, reaching an average belonging to the high level. Statistically significant differences were also found in the high level, which decreased from 11.63 (±1.25) to 10.00 (±2.63). In this case, although the average was lower, it still belonged to the high level. Considering these results, the intervention helped the participants of all levels to reach a mean near the high level, confirming the previous results of Table 6.

Regarding the second research question, *does gender influence the CT skills of pre-service teachers before and after an ER-based intervention under a science and mathematics teaching approach?* the results indicated a higher mean CT level in males (8.95 ± 2.38) than in females (7.71 ± 2.49) before the intervention (Table 2). These differences (Table 4) were statistically significant, which is consistent with previous studies [28,39].

Regarding the results after the intervention, it was observed (Table 4) that there were no statistically significant differences participants (*p* = 0.86) between the male (9.71 ± 2.87) and female participants (9.60 ± 2.72). The results of the Wilcoxon test (Table 5) showed that there were no significant differences for the male participants, but there were for the female participants. This evidence shows that the global increase in the level of CT was essentially caused by the increase in the level of CT in the female participants. It is important to clarify that, despite the lack of statistically significant differences, the male participants also showed increases in the different CT skills examined.

This finding is in accordance with the scientific literature [6], which shows that females, despite having a lower predisposition and CT skills prior, after appropriate interventions, develop a level of CT that is equivalent to or higher than that of males. Our results are also in agreement with the study of Günbatar and Bakırcı [30], in which no significant differences in CT were found between males and females after interventions, so ER may be a tool that allows for reducing gender differences in the development of these skills.

The CT scores observed (Table 6 and Table 7) are consistent with these findings, with more women (75.64%) than men (63.16%) demonstrating both intermediate and lower CT scores before the intervention. Conversely, males had a higher presence at thehigh level (36.84%) than females (17.95%). However, both women (44.87%) and men (42.11%) were equally represented at the middle and high levels (53.85% and 55.26%) after the intervention. In both genders, there was a statistically significant difference between their pre- and post-intervention mean scores, with a mean close to the high level, regardless of gender and initial score. Moreover, this is complemented by the results obtained in terms of concepts and sub-concepts, since the results showed that there was a higher mean for men in 11 of the 14 items of the questionnaire. On the post-test, however, the difference was more pronounced, with males scoring higher on seven items and females on five items.

Our findings help to fill the gap regarding the development of CT literacy among pre-service teachers by providing robotics-based training and learning activities that focus on science and math learning. The results indicated that the female gender significantly increased their CT skills, matching the skills of the male gender. This fact shows that CT skills, understood as one of the necessary skills for people today, can be promoted and developed through interventions based on ER and this approach. Therefore, there is a call for more interventions of this nature to reduce the gender gaps that may exist in certain areas such as computer science and scientific and mathematical fields, especially when it involves pre-service teachers.

## 5. Conclusions

The results of this study indicate that the integration of Educational Robotics into science and mathematics education can be an effective approach to the development of Computational Thinking (CT) skills in pre-service teachers. The main results showed a significant increase in CT skills between the pre-test (8.11) and the post-test (9.63), highlighting sub-concepts such as *Simple Functions*, *While* and *Compound Conditional*, which are highly complex skills. In addition, an increase in the percentage of the sample belonging to the high level of Computational Thinking stood out, increasing from 24.14% in the pre-test to 54.31% in the post-test.

However, although encouraging, these results should be viewed with caution. Despite the overall improvement in CT skills, some subconcepts, such as *Repeat until* and *Simple conditionals*, did not show a comparable degree of improvement. This observation suggests that, while Educational Robotics in this approach had a positive impact on CT skill development, not all sub-concepts showed the same level of progress. This underscores the importance of supporting this strategy with complementary or specific approaches to strengthen those sub-concepts that did not show a significant degree of improvement.

With respect to gender, the results found significant differences before the intervention, with higher mean CT skills observed in males compared to in females. However, these differences between males and females were equalised after the intervention. Also, the percentage of the sample belonging to the high level (53.85% in females and 55.26% in males) was similar in both genders, and the level was equalised regardless of the starting level of each gender. Nevertheless, it is important mention that some specific sub-concepts still showed some differences, suggesting that strategies need to be refined in order to ensure a uniform improvement in all areas of Computational Thinking, regardless of gender.

Therefore, the focus on teaching science and mathematics through the Educational Robotics interventions allows for increasing the development of the Computational Thinking skills of pre-service teachers, while mitigating gender differences, allowing for equalising the level of skills developed.

## 6. Limitations of the Study and Future Research Lines

One of the main factors is the sample of the study, which shows an unbalanced representation of men and women, due to the educational reality of the university context. Another limitation is the fact that the research was not carried out with a control group, which would make it possible to distinguish and compare the results found. Furthermore, studies are needed that examine how these improvements are preserved over time or whether the intervention has the same effect in the long term for both male and female participants. Interesting results that would add further evidence to the results of the present study could be obtained by longitudinal studies or by administering several post-intervention tests over time.

Future lines of research could analyse the level of quality of the learning by the means of taxonomies that make it possible to relate these skills to the level of cognitive complexity and, thus, to determine the level of thinking at which they are found.

## Figures and Tables

**Table 1:** Description of the training program.

Session	Session Content	Duration
Pre-Session	Pre-test Questionnaire Computational Thinking	30 min
Session 1	Introduction to Scratch 3.0 software and basic notions of block programming	30 min
	Using Scratch to teach geometric content in primary education	30 min
Session 2	Mandala challenge and learning feedback	60 min
Session 3	Introduction to Educational Robotics as a teaching tool in primary education (basic concepts)	20 min
	Experimentation with Mind Designer^®^ Robotics Kit and App (basic functions)	20 min
	Using Mind Designer^®^ to teach science and mathematics content	20 min
Session 4 and 5	Robotic board challenge and learning feedback	120 min
Post-Session	Post-test Questionnaire Computational Thinking	30 min

**Table 2:** Descriptive results of the pre-test questionnaire on Computational Thinking.

Computing Concept	Sub-Concept	Item	Pre-Test					
			M		F		T	
			X	SD	X	SD	X	SD
**Addresses**	Addresses	1	1.00	0.00	0.83	0.37	0.89	0.32
		2	0.61	0.50	0.69	0.47	0.66	0.47
Loops	Repeat	3	0.84	0.37	0.77	0.42	0.79	0.41
		4	0.47	0.51	0.36	0.48	0.40	0.49
	Repeat until	5	1.00	0.00	0.97	0.16	0.98	0.13
		6	0.13	0.34	0.32	0,47	0.26	0.44
Conditional	Simple conditional	7	0.81	0.39	0.64	0.48	0.70	0.46
		8	0.47	0.51	0.31	0.46	0.36	0.48
	Compound conditional	9	0.71	0.46	0.59	0.50	0.63	0.49
		10	0.63	0.49	0.49	0.50	0.53	0.50
	While	11	0.63	0.49	0.46	0.50	0.52	0.50
		12	0.18	0.39	0.30	0.46	0.26	0.44
Functions	Simple functions	13	0.79	0.41	0.64	0.48	0.69	0.47
		14	0.63	0.49	0.36	0.48	0.45	0.50
		**Total**	**8.95**	**2.38**	**7.71**	**2.49**	**8.11**	**2.52**

**Table 3:** Descriptive results of the post-test questionnaire on Computational Thinking.

Computing Concept	Sub-Concept	Item	Pre-Test					
			M		F		T	
			X	SD	X	SD	X	SD
Addresses	Addresses	1	0.92	0.27	0.90	0.31	0.90	0.30
		2	0.74	0.45	0.80	0.42	0.80	0.41
Loops	Repeat	3	0.87	0.34	0.78	0.41	0.80	0.41
		4	0.50	0.51	0.51	0.50	0.51	0.50
	Repeat until	5	0.97	0.16	0.95	0.22	0.95	0.22
		6	0.40	0.50	0.27	0.45	0.27	0.45
Conditional	Simple conditional	7	0.74	0.45	0.73	0.45	0.73	0.45
		8	0.50	0.51	0.37	0.49	0.37	0.49
	Compound conditional	9	0.76	0.43	0.73	0.45	0.73	0.45
		10	0.76	0.43	0.62	0.49	0.63	0.49
	While	11	0.55	0.50	0.82	0.39	0.82	0.39
		12	0.40	0.50	0.44	0.50	0.47	0.50
Functions	Simple functions	13	0.95	0.23	0.90	0.31	0.90	0.31
		14	0.66	0.48	0.67	0.47	0.68	0.47
		**Total**	**9.71**	**2.87**	**9.60**	**2.72**	**9.63**	**2.75**

**Table 4:** Mann–Whitney U test by gender in the pre-test and post-test of the CTt.

Computing Concept	Sub-Concept	Item		U Mann Whitney Test				
			Pre-Test			Post-Test		
			S	*p*	*ES*	S	*p*	*ES*
Addresses	Addresses	1	1235	0.01 *	0.16667	1447	0.69	0.02362
		2	1353	0.36	0.08704	1396	0.49	0.04521
Loops	Repeat	3	1374	0.37	0.07287	1373	0.34	0.08637
		4	1312	0.24	0.11471	1463	0.90	0.01282
	Repeat until	5	1444	0.33	0.02564	1445	0.54	0.02497
		6	1202	0.03 *	0.18893	1296	0.17	0.12551
Conditional	Simple conditional	7	1079	0.06	0.17476	1473	0.95	0.00607
		8	1236	0.08	0.16599	1292	0.19	0.12821
	Compound conditional	9	1303	0.21	0.12078	1434	0.71	0.03239
		10	1268	0.15	0.14440	1263	0.15	0.14777
	While	11	1230	0.09	0.17004	1085	0.00 *	0.26788
		12	1318	0.21	0.11066	1421	0.42	0.04116
Functions	Simple functions	13	1262	0.11	0.14845	1408	0.37	0.04993
		14	1078	0.01 *	0.27260	1469	0.37	0.00877
		**Total**	**1069**	0.01 *	**0.27901**	**1457**	**0.86**	**0.01721**

* Statistically significant differences (*p* < 0.05).

**Table 5:** Wilcoxon test in males, females, and total CTt.

Computing Concept	Sub-Concept	Item	Wilcoxon Test								
			M			F			T		
			S	*p*	*ES*	S	*p*	*ES*	S	*p*	*ES*
Addresses	Addresses	1	6.0	0.15	1.00	88.0	0.28	−0.24	137.5	0.70	−0.08
		2	40.0	0.21	−0.33	259.0	0.19	−0.20	490.0	0.07	−0.24
Loops	Repeat	3	20.0	0.79	−0.11	188.5	0.72	−0.03	323.0	0.63	−0.05
		4	56.0	0.82	−0.07	192.5	0.04 *	−0.35	450.0	0.06	−0.27
	Repeat until	5	1.00	1.00	1.00	14.0	0.49	0.33	20.0	0.30	0.43
		6	15.0	0.01 *	−0.71	409.5	0.52	0.11	609.5	0.41	−0.12
Conditional	Simple conditional	7	42.0	0.39	0.27	285.0	0.25	−0.19	539.0	0.57	−0.08
		8	42.0	0.81	−0.08	340.0	0.43	−0.13	609.5	0.41	−0.12
	Compound conditional	9	59.5	0.64	−0.13	216.0	0.06	−0.31	494.0	0.07	−0.25
		10	18.0	0.15	−0.45	315.0	0.09	−0.24	477.0	0.04 *	−0.28
	While	11	110.0	0.51	0.16	74.0	<0.001 *	−0.78	420.0	<0.001 *	−0.45
		12	13.0	0.02 *	−0.67	266.5	0.03 *	−0.27	397.5	0.01 *	−0.36
Functions	Simple functions	13	11.0	0.07	−0.60	77.5	<0.001 *	−0.67	143.5	<0.001 *	−0.65
		14	72.0	0.83	−0.06	114.0	<0.001 *	−0.63	385.0	<0.001 *	−0.45
		**Total**	**188.8**	**0.16**	**−0.29**	**626.5**	<0.001 *	**−0.50**	**1457.5**	<0.001 *	**−0.45**

* Statistically significant differences (*p* < 0.05)

**Table 6:** Frequencies and percentages according to levels of Computational Thinking by gender in pre-test and post-test.

Gender	Level	Pre-Test		Post-Test	
		n	%	n	%
**Female (n = 78)**	Under	5	6.41%	1	1.28%
	Medium	59	75.64%	35	44.87%
	High	14	17.95%	42	53.85%
					
**Male (n = 38)**	Under	0	0.00%	1	2.63%
	Medium	24	63.16%	16	42.11%
	High	14	36.84%	21	55.26%
					
**Total (n = 116)**	Under	5	4.31%	2	1.72%
	Medium	83	71.55%	51	43.97%
	High	28	24.14%	63	54.31%

**Table 7:** Averages and Wilcoxon test of averages on Computational Thinking levels and gender.

Gender	Level	Pre-Test			Post-Test			Wilcoxon Test	
		n	X	SD	n	X	SD	*p*	*ES*
**Female (n = 78)**	Under	5	3.00	0.71	5	10.40	2.07	0.06	−1.00
	Medium	59	7.15	1.38	59	9.46	2.87	<0.001 *	−0.68
	High	14	11.77	1.01	14	9.92	2.25	0.02 *	0.84
									
**Male (n = 38)**	Under	0	0.00	0.00	0	0.00	0.00	NaN	NaN
	Medium	24	7.46	1.29	24	9.50	2.81	0.00 *	−0.51
	High	14	11.50	1.45	14	10.50	3.05	0.07	0.53
									
**Total (n = 116)**	Under	5	3.00	0.71	5	10.40	2.07	0.06	−1.00
	Medium	83	7.24	1.35	83	9.47	1.35	<0.001 *	−0.64
	High	28	11.63	1.25	28	10.00	2.63	0.00 *	0.74

* Statistically significant differences (*p* < 0.05).

## Data Availability

The original contributions presented in this study are included in the article. Further inquiries can be directed to the corresponding author.
